# Dual Roles of NIX/BNIP3L in Tumors: Friend or Foe

**DOI:** 10.3390/biology15040302

**Published:** 2026-02-09

**Authors:** Fanghui Ge, Jingxuan Shu, Ziqian Liu, Hong Zhang, Jiandong Wang

**Affiliations:** 1 Sichuan Provincial Engineering Laboratory for Prevention and Control Technology of Veterinary Drug Residue in Animal-Origin Food, School of Laboratory Medicine, Chengdu Medical College, Chengdu 610500, China; 202022038@cmc.edu.cn (F.G.);; 2 Experiment Teaching Demonstration Center of Laboratory Medicine, School of Laboratory Medicine, Chengdu Medical College, Chengdu 610500, China; 3 Department of Pediatrics, School of Clinical Medicine, Chengdu Medical College, Chengdu 610500, China; 18381084711@163.com; 4 Chengdu Medical College Office of Science and Technology, Chengdu Medical College, Chengdu 610500, China; 5 Key Laboratory of Nuclear and Radiation Damage Mechanisms and New Treatment Technology at Chengdu Medical College of Sichuan Province, Chengdu 610500, China

**Keywords:** NIX/BNIP3L, mitophagy, oxidative stress, tumors, cell death

## Abstract

Cancer is one of the leading causes of disease-related death worldwide, and targeting key regulatory genes to induce programmed cell death in tumor cells has emerged as a crucial therapeutic strategy, following surgery, radiotherapy, and chemotherapy. As a mitochondrial outer membrane protein, NIX/BNIP3L can both mediate apoptosis to inhibit tumor cell growth and promote tumor cell survival by clearing intracellular reactive oxygen species (ROS) through mitophagy. Therefore, we summarize a brief overview of the structure and function of NIX/BNIP3L, as well as the mechanisms of NIX/BNIP3L generation and degradation, the role of NIX/BNIP3L in mediating apoptosis and mitophagy and to advance the understanding of the roles of NIX/BNIP3L in glioblastoma, lung cancer, hepatocellular carcinoma, breast cancer, pancreatic cancer, colorectal cancer and hematologic neoplasms, aiming to enhance treatment precision and improve patient outcomes.

## Simple Summary

As a mitochondrial outer membrane protein, NIX/BNIP3L not only mediates apoptosis to inhibit tumor growth, but also promotes tumor cell survival by eliminating intracellular ROS via mitophagy. Through a systematic summary of the gene structure of NIX, the pathways of biogenesis and degradation, the mechanisms by which it orchestrates apoptosis and mitophagy, and the roles in glioblastoma, lung cancer, hepatocellular carcinoma, breast cancer, pancreatic cancer, colorectal cancer, and hematological malignancies, we anticipate providing a novel theoretical foundation for the personalized treatment and prognosis of cancer patients.

## 1. Introduction

Molecular-targeted therapy, recognized as the fourth major modality in cancer treatment following surgery, radiotherapy, and chemotherapy, constitutes a crucial component of molecular medicine. Molecular targeted therapies achieve anticancer effects through various mechanisms, such as inhibition of cell proliferation, metastasis, angiogenesis, and induction of apoptosis, enabling precise eradication of cancer cells while minimizing damage to normal tissues [1]. This high selectivity significantly spares normal cells, leading to a decrease in common adverse reactions associated with traditional chemotherapy, such as myelosuppression and severe alopecia, thereby substantially improving patients’ quality of life and treatment tolerance. For instance, imatinib, a tyrosine kinase inhibitor, achieves an eight-year survival rate of 87% in chronic myeloid leukemia, markedly superior to the outcomes of traditional chemotherapy [2].

However, tumor heterogeneity (encompassing spatial, temporal, and cellular dimensions) also leads to substantial variations in molecular characteristics across different regions of a tumor and at different time points, adversely affecting treatment efficacy [3]. Furthermore, only approximately 20–40% of cancer patients harbor well-defined, actionable driver gene mutations [4]. A significant number of tumors, such as certain pancreatic cancers, triple-negative breast cancers, and lung squamous cell carcinomas, lack effective targets and thus the patients cannot benefit from existing targeted therapies. Therefore, developing novel therapeutic targets may hold promise for enabling more patients to benefit from targeted treatment strategies [5].

NIX (B-cell leukemia/lymphoma 2 (Bcl-2)/adenovirus E1B 19 kDa interacting protein 3-like, also called BNIP3L) was initially discovered through yeast two-hybrid screening for cellular proteins interacting with E1B 19K and subsequent sequencing in a human placental cDNA library. NIX/BNIP3L contains the apoptotic effector domain (Bcl-2 homology 3, BH3); as a result, it was classified as a BH3-only protein early [6,7,8]. Unlike other Bcl-2 family proteins, NIX/BNIP3L can paradoxically promote cell survival under certain conditions. For instance, in acute myeloid leukemia (AML) cells, deficiency in BNIP3L has been shown to increase their sensitivity to mitochondria-targeting agents [9].

NIX is also a mitochondrial membrane protein that integrates into the outer mitochondrial membrane (OMM) via its conserved C-terminal transmembrane domain. Through its N-terminal LC3-interacting region (LIR) motif, it binds to processed LC3, thereby facilitating the initiation of mitophagy [10]. Mitophagy is a selective form of autophagy responsible for clearing dysfunctional or superfluous mitochondria, thereby maintaining mitochondrial homeostasis within the cell [11]. Under physiological stress conditions, such as hypoxia and nutrient deprivation, the expression of BNIP3 and NIX can be induced to promote mitochondrial turnover. In tumor cells, the dysregulated expression of BNIP3 and NIX is associated with tumor growth. Moreover, the NIX/BNIP3L gene is localized to the autosomal chromosomal region 8p21 [7,12,13]. This region frequently exhibits loss of heterozygosity in multiple tumor types [13], suggesting that this recurrent loss of heterozygosity indicates NIX/BNIP3L plays a critical role in tumorigenesis and cancer progression. This review provides a comprehensive assessment of the role of NIX/BNIP3L in tumor initiation and progression by summarizing its molecular architecture, biogenesis, and degradation pathways, mechanisms in mediating apoptosis, and regulating mitophagy. This synthesis not only extends our understanding of NIX/BNIP3L but also aids in developing more effective and personalized therapeutic strategies for various solid and non-solid tumors.

## 2. Functional Domains of NIX/BNIP3L

**Figure 1:**
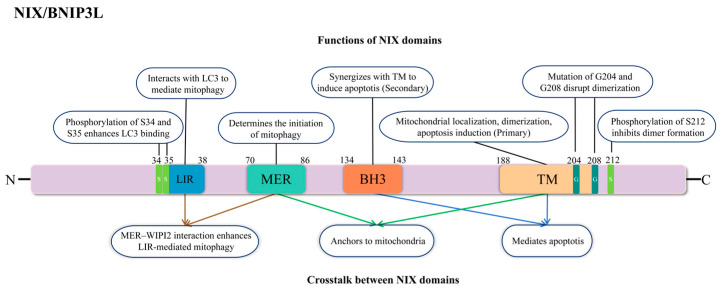
Schematic diagram of NIX domain functions. The LIR domain interacts with LC3 to mediate mitophagy; the BH3 and TM domains cooperate to induce apoptosis, with the TM domain also involved in dimerization. Key regulatory sites include the S34/S35 phosphorylation sites that enhance LC3 binding and the G204/G208 mutations that disrupt dimerization. Additionally, the MER domain interacts with WIPI2 to enhance LIR-mediated mitophagy. Arrows in the schematic illustrate the functional interactions and regulatory relationships between these domains.

NIX/BNIP3L can not only induce apoptosis but also mediate mitophagy. It has structural domains with four biological functions, namely the transmembrane structural domain (TM), Bcl-2 homologue 3 (BH3), the minimal essential region (MER), and the microtubule-associated protein 1 light chain 3-interacting region (LIR) [14]. The TM domain located in the C-terminal region, which includes the membrane-anchoring segment, is essential for mitochondrial targeting and dimerization of BNIP3L [14,15], targeting mitochondria to induce membrane potential loss and cytochrome c (Cyt c) release. Furthermore, BNIP3L competes with Beclin 1 for binding to Bcl-2 and Bcl-XL, thereby disrupting Beclin 1–Bcl-2/Bcl-XL complexes, releasing Beclin 1 and promoting autophagy [16]. Unlike canonical pro-apoptotic BH3-only proteins, the BH3 domain of NIX/BNIP3L is not essential for its ability to induce cell death [15,17]. The MER domain comprises three consecutive hydrophobic amino acid residues flanked by charged residues. This domain is essential for NIX-mediated mitophagy. Ji Zhang et al. demonstrate that mutation of the central leucine residue within MER abolishes NIX activity and prevents the rescue of impaired mitochondrial clearance [10,18]. NIX contains LIR that is oriented toward the cytoplasm, enabling the recruitment of LC3 proteins to the autophagosome. A study found that the deletion of the LIR domain in NIX (amino acids 150–171) abolished its ability to activate mitophagy [19]. All in all, the TM, BH3, MER, and LIR domains of NIX/BNIP3L function through a highly coordinated mechanism. The TM domain provides essential mitochondrial targeting and dimerization. Although atypical, the BH3 domain participates in the complex interaction network of the Bcl-2 family [17]. The MER may act as an upstream signal integrator to initiate mitophagy, while the LIR motif is directly responsible for recruiting autophagosomes and executing mitochondrial clearance. The interplay among these domains ensures that NIX/BNIP3L can respond to cellular stress signals, precisely regulate mitophagy, and thereby maintain intracellular homeostasis under stress conditions (Figure 1).

## 3. Generation and Degradation of NIX/BNIP3L

The expression of NIX is controlled at multiple levels—transcription, translation, post-translational modification, and environmental responses. Meanwhile, Hypoxia inducible factor-1 (HIF-1) induces apoptosis by upregulating recombinant Bcl2/Adenovirus E1B 19kDa interacting protein 3 (BNIP3) and BNIP3L [20,21]. Under hypoxic conditions, the activity of prolyl hydroxylases (PHDs), which hydroxylate HIF-1α, targeting it for degradation, is inhibited. Following stabilization and cytoplasmic accumulation [22], the HIF-1 complex translocates into the nucleus. There, it binds to the hypoxia response element (HRE), activating transcription of the BNIP3L gene [23]. Furthermore, hypoxia is not the sole condition that induces NIX expression [21], as its regulatory mechanism also involves other transcription factors.

In addition, NIX expression is regulated by the PI3K/AKT/mTOR and mitogen-activated protein kinase (MAPK) signaling pathways. AKT (serine/threonine kinase or protein kinase B) is mainly regulated by phosphatidylinositol tri-kinase (PI3K), which recruits AKT to the cell membrane and phosphorylates it at the Thr308 and Ser473 sites, subsequently phosphorylating a variety of downstream target proteins to promote cell proliferation, survival, and metastasis [24]. AKT phosphorylates forkhead box O3a (FoxO3a), leading to its cytoplasmic sequestration, inhibition of transcriptional activity, and subsequent suppression of BNIP3L transcription [25]. FoxO3a, an important transcription factor, is localized to chromosome 6q2 and binds to the promoter of NIX to induce NIX transcription. In this study, they also showed that BNIP3L is a downstream target of FoxO3a [26]. Similarly, upregulation of NIX was likewise observed in AKT-inactivated lung cancer cells [27].

Mammalian target of rapamycin (mTOR) is a downstream target of AKT that negatively regulates NIX. AKT phosphorylates and inhibits tuberous sclerosis complex 1/2, resulting in mTORC1 activation and autophagy inhibition [28]. Similarly, mTORC2 can directly phosphorylate AKT to enhance its activity, thus forming a positive feedback loop [29]. NIX-mediated mitophagy is similarly inhibited by activation of the AKT/mTOR pathway, and autophagy is further down-regulated, occurring as a result of inhibition of the expression of multiple autophagy proteins such as NIX [30,31,32,33]. However, mTOR inhibition of NIX and its mediated autophagy remains to be explored. Interestingly, previous studies have shown that mTORC1 activation depends on lysosomal GTPases such as Ras homolog enriched in brain (RHEB), which can promote mitophagy in a BNIP3L-dependent manner [30,34], but the reasons why did not reverse the onset of NIX-mediated mitophagy remain to be explored.

The MAPK pathway positively regulates NIX through the activation of p38 mitogen-activated protein kinase (p38MAPK) and JNK. NIX forms a complex with c-Jun N-terminal kinase (JNK) via the plethora of SH3s (POSH) scaffold, enabling reciprocal stabilization and protection from degradation. This complex activates p53, thereby initiating the BAX-dependent apoptotic pathway. Phosphorylation of p38MAPK promotes NIX expression, induces NIX-LC3II-mediated mitophagy, and increases apoptosis in human osteosarcoma cells [11]. JNK binds to NIX, upregulating NIX expression and promoting NIX-mediated mitophagy and apoptosis. Xing Zhou et al. reported that diltiazem hydrochloride protects against myocardial ischemia/reperfusion injury through an NIX-mediated mechanism both in vivo and in vitro [35]. This JNK-induced NIX-dependent mitophagy model was also validated in cancer-associated fibroblasts (CAFs) within the breast cancer microenvironment [36].

Intracellular protein degradation primarily occurs through two major pathways: the autophagy-lysosome pathway and the ubiquitin-proteasome pathway. In experiments conducted by Wu X et al., treatment with lysosomal inhibitors and proteasomal inhibitors to block these two pathways, respectively, revealed that lysosomal inhibition failed to restore NIX expression, whereas proteasomal inhibition significantly upregulated the expression of NIX. This suggests that NIX degradation is predominantly dependent on the ubiquitin-proteasome pathway [37].

Upon restoration of mitochondrial function or under conditions of NIX overaccumulation, ubiquitin molecules are activated by the E1 ubiquitin-activating enzyme. The activated ubiquitin is then transferred from E1 to an E2 ubiquitin-conjugating enzyme, forming an E2-ubiquitin complex. Subsequently, an E3 ubiquitin ligase recognizes NIX and facilitates the transfer of ubiquitin from the E2 enzyme onto NIX. This ubiquitination marks NIX for degradation, leading to its recognition and breakdown by the 26S proteasome. The 26S proteasome is composed of a 20S core particle and a 19S regulatory particle. The 20S core is a complex containing proteolytic catalytic sites, while the 19S regulatory complex comprises several ATPase subunits and other subunits, which are likely involved in the specific recognition of ubiquitinated proteins by the 26S proteasome. The 26S proteasome, via its 19S regulatory particle, recognizes the ubiquitinated NIX protein, translocates it into the 20S core, where NIX is degraded into short peptide fragments. These peptides are ultimately released back into the cytoplasm for reuse.

**Figure 2:**
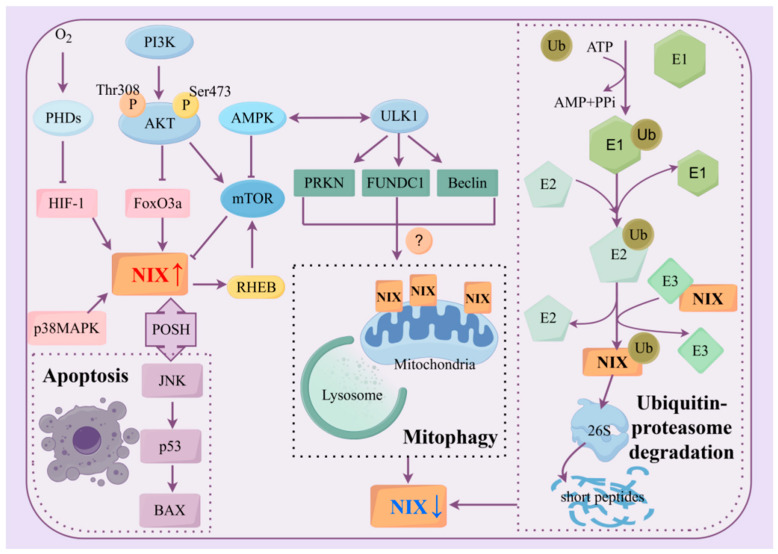
Pathways of NIX protein degradation and NIX production. The ubiquitin-proteasome pathway is through ubiquitination of modified NIX, recognized by the proteasome 26S and degraded to short peptides; the autophagy lysosome pathway is through the promotion of PRKN, FUNDC1, and Beclin-mediated mitochondrial autophagy via ULK1 in the AMPK-activated state (A question mark in the schematic diagram represents the authors’ conclusions based on the synthesis of the reported literature, and whether NIX is degraded through this pathway needs to be proved by more experiments). HIF-1, FoxO3a, p38MAPK, and JNK promote the production of NIX proteins, and JNK promotes NIX protein expression by stabilizing the binding of POSH proteins. In addition, NIX protein production is inhibited by mTOR, but in turn, NIX expression promotes mTOR expression through RHEB. Red upward arrows indicate the generation of NIX, and blue downward arrows indicate its degradation. Abbreviations: AKT: serine/threonine kinase; AMPK: Adenosine 5′-monophosphate (AMP)-activated protein kinase; BAX: BCL2-Associated X protein; E1: Ubiquitin-activating enzyme; E2: Ubiquitin-Conjugating Enzyme; E3: Ubiquitin-ligase enzyme; FoxO3a: Forkhead box O3a; FUNDC1: FUN14 domain-containing protein 1; HIF-1: Hypoxia inducible factor-1; mTOR: Mammalian Target of Rapamycin; p38MAPK: p38 mitogen-activated protein kinase; PHDs: Prolyl hydroxylases; PI3K: Phosphoinositide 3-Kinase; POSH: Plenty of SH3s; PRKN: E3 ubiquitin-protein ligase parkin; RHEB: Ras homolog enriched in brain; Ub: Ubiquitin; ULK1: Unc-51 like autophagy activating kinase 1.

Furthermore, under conditions of nutrient or energy deficiency, the activation of AMP-activated protein kinase (AMPK), a negative regulator of mTOR, induces autophagy. In this context, NIX expression shows a negative correlation with mitophagy [38,39]. This may be because AMPK signaling regulates mitophagy through pathways mediated by PRKN and FUNDC1, rather than through the NIX-dependent mitophagy pathway [39]. Consequently, NIX, which does not play a role in this specific form of mitophagy, is reduced as an outer mitochondrial membrane protein following the extensive clearance of mitochondria (Figure 2).

## 4. Mechanism of NIX-Mediated Apoptosis

Apoptosis is a highly regulated form of programmed cell death, comprising intrinsic and extrinsic pathways. The extrinsic pathway, also termed the death receptor pathway, is a programmed death process directly activated by extracellular death signals such as cytokines or immune molecules. NIX primarily regulates the intrinsic apoptosis pathway, the mitochondrial apoptosis pathway, promoting cell death by disrupting mitochondrial function and integrity. Upon induction of apoptosis, NIX transcription is activated, leading to elevated protein levels. NIX relies on its C-terminal transmembrane domain for localization to the mitochondrial outer membrane. At this site, NIX binds to cytochrome c anti-apoptotic proteins Bcl-2 and Bcl-XL, antagonizing their inhibitory effect on the pro-apoptotic proteins BAK and BAX. Consequently, BAK and BAX oligomerize within the mitochondrial outer membrane, increasing its permeability and triggering the release of Cytc [40]. Subsequently, the released Cytc binds to apoptotic protease activating factor 1 (Apaf-1) and ATP, forming the apoptosome. This apoptosome then activates the caspase cascade, ultimately leading to apoptosis [41]. Moreover, during apoptosis potentiation by the JNK cascade, the scaffold protein POSH binds to NIX, and these proteins mutually stabilize each other, preventing their degradation and disruption by JNK [42]. Importantly, BNIP3L/NIX-induced apoptosis is mechanistically distinct from that triggered by canonical BH3-only proteins. The pro-apoptotic function of BNIP3L/NIX does not strictly rely on its BH3 domain, and the process is dependent on the opening of the mitochondrial permeability transition pore (MPTP).

**Figure 3:**
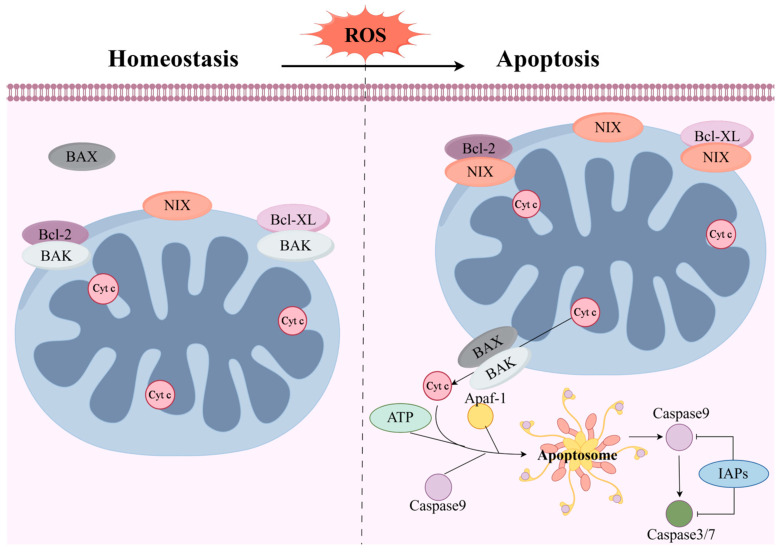
Mechanism of NIX-mediated apoptosis. After the cell receives an external stimulus to initiate apoptosis, NIX binds to Bcl-2 and Bcl-XL, causing BAK and BAX to form oligomerization pores at the outer mitochondrial membrane, releasing cytochrome c (Cyt c). Subsequently, Cyt c, Apaf-1, and ATP form the apoptosome, which activates caspase-9. Activated caspase-9 then cleaves procaspase-3/7 to generate active caspase-3/7, thereby triggering apoptosis. The activity of caspase-3, -7, and -9 is inhibited by IAPs. Abbreviations: Cyt c: Cytochrome c; Apaf-1: Apoptotic protease-activating factor 1; IAPs: Inhibitor of Apoptosis Proteins.

In contrast, typical BH3-only proteins primarily promote cytochrome c release and initiate an MPTP-independent apoptotic pathway by engaging anti-apoptotic Bcl-2 family proteins via their BH3 domain. BNIP3L/NIX predominantly dimerizes via its C-terminal transmembrane domain and anchors itself to the outer mitochondrial membrane. This integration disrupts mitochondrial membrane integrity, ultimately leading to apoptosis [17]. Furthermore, the p75 neurotrophin receptor (p75NTR) likewise promotes apoptosis by binding to and stabilizing NIX, thereby inducing activation of the JNK-p53-BAX pathway. Specifically, under neuronal stress conditions, p75NTR directly binds to both the transmembrane domain and the BCopper domain of NIX, stabilizing and upregulating NIX expression. The resulting complex further recruits POSH, a scaffold protein for JNK, thereby leading to sustained JNK signaling activation. The activated JNK subsequently phosphorylates and activates the transcription factor p53, which ultimately promotes the upregulation and mitochondrial translocation of the pro-apoptotic protein BAX, driving mitochondrial-dependent apoptosis. This signaling axis is markedly induced in neurons following intracerebral hemorrhage (ICH), and its activation level closely correlates with the extent of cellular apoptosis, suggesting that the POSH-JNK-p53-BAX axis constitutes a decisive pathway mediating neuronal apoptosis after ICH. This discovery not only reveals a novel regulatory mechanism for neuronal apoptosis post-ICH but also provides potential therapeutic targets for neuroprotective strategies aimed at this pathway [8] (Figure 3).

## 5. Mechanism of NIX-Mediated Mitophagy

The molecular mechanisms of mitophagy are primarily categorized into receptor-dependent and ubiquitin-dependent pathways. The receptor-dependent pathway involves direct binding of autophagy receptor proteins (e.g., NIX) to LC3 to target mitochondria for autophagic clearance. The ubiquitin-dependent pathway, represented by the PINK1/Parkin pathway, entails ubiquitin-mediated tagging of damaged mitochondria, which subsequently recruits autophagy adaptor proteins like Parkin to facilitate their degradation. Notably, NIX is capable of mediating mitophagy through both the receptor-dependent and ubiquitin-dependent pathways.

In the NIX-mediated receptor-dependent pathway, NIX relies on its C-terminal transmembrane domain for localization to mitochondria. Its N-terminal LIR then binds to LC3/γ-Aminobutyric acid (GABA) type A receptor-associated protein (GABARAP) proteins on the phagophore membrane, recruiting the autophagosome to target mitochondria [19]. This process enables mitochondrial clearance independently of ubiquitination. Furthermore, phosphorylation of NIX constitutes a critical regulatory mechanism for NIX-mediated mitophagy. Intriguingly, phosphorylation at distinct sites exerts opposing effects: Phosphorylation of Ser34 and Ser35 residues within the N-terminal region activates NIX, enhancing its binding to LC3B and thereby promoting mitochondrial clearance [43]. Conversely, phosphorylation of the C-terminal serine residue 212 inhibits homodimer formation, consequently suppressing mitophagy [14]. Notably, studies have found that NIX binding to LC3B is relatively weaker compared to its binding to LC3A and GABARAP proteins, suggesting that LC3A and GABARAP play a dominant role in this pathway [43]. Moreover, phosphorylation modification and dimer formation co-regulate the efficiency of NIX-mediated mitochondrial autophagy. BNIP3L^G204A^ or BNIP3L^G208V^ mutations in the BNIP3L transmembrane domain would disrupt its dimerization [14].

**Figure 4:**
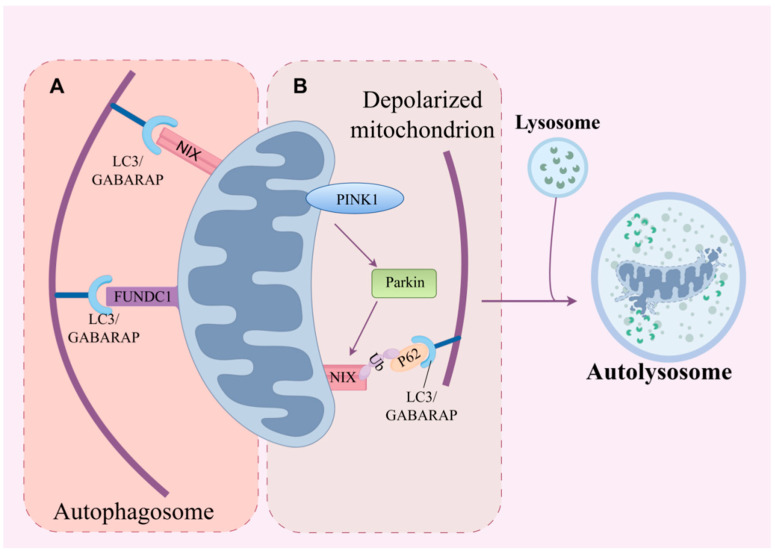
Two pathways of NIX-mediated mitophagy. (**A**) Receptor-dependent pathway: Mitophagy receptors such as NIX and FUNDC1 clear mitochondria by directly binding to members of the Atg8 family of proteins (e.g., LC3, GABARAP). (**B**) Ubiquitination-dependent pathway: Mitophagy is mediated by ubiquitination of mitochondrial proteins via the PINK1/Parkin pathway following a decrease in mitochondrial membrane potential. This process depends on autophagy adaptor proteins binding to LC3. Abbreviations: FUNDC1: FUN14 domain-containing protein 1; LC3: Microtubule-associated protein 1A/1B light chain 3; GABARAP: Gamma-aminobutyric acid receptor-associated protein; PINK1 Pink1: PTEN-induced putative kinase protein 1; Parkin: Parkinson’s disease protein.

The NIX-mediated ubiquitin-dependent pathway primarily utilizes the PINK1/Parkin cascade, where ubiquitination of NIX facilitates mitophagy. PTEN-induced putative kinase 1 (PINK1) is a mitochondrial-targeted kinase. In normal mitochondria, PINK1 is imported into the inner membrane and subsequently degraded. However, upon mitochondrial damage and loss of membrane potential (ΔΨm), PINK1 accumulates on the outer mitochondrial membrane [44]. The ubiquitin-ligase enzymes (E3) ubiquitin ligase Parkin then ubiquitinates NIX, enabling its interaction with the autophagy adaptor protein SQSTM1/p62 [45]. Subsequently, Parkin, via its own LIR domain, binds to LC3 proteins. This dual interaction recruits the autophagosome to damaged mitochondria, ultimately leading to lysosomal fusion and mitochondrial degradation [46]. Intriguingly, studies have revealed that in SQSTM1/p62-deficient cells, mitophagy via the ubiquitin-dependent NIX pathway can still occur, mediated by the alternative adaptor protein neighbor of BRCA1 gene 1 (NBR1) [47] (Figure 4).

## 6. Role of NIX-Mediated Apoptosis and Mitophagy in Tumors

### 6.1. Glioblastoma

Glioblastoma (GBM), the most prevalent and aggressive primary brain malignancy, demonstrates remarkably poor treatment outcomes. Current standard-of-care involving maximal safe resection followed by radiotherapy and temozolomide chemotherapy achieves only a median survival of 12–14 months, with 2-year survival rates remaining at 15–26%. These sobering statistics have shown minimal improvement over recent decades, despite significant advances in both clinical and basic neuroscience research [30]. NIX mRNA levels are tumor grade-dependent and higher in glioblastoma. In low-grade gliomas (e.g., astrocytoma), where NF-κB expression is minimal, NIX may primarily function as a tumor suppressor by promoting BAX-dependent apoptosis. Conversely, in aggressive gliomas (such as GBM), elevated NIX expression and NF-κB pathway activation drive its oncogenic role, favoring tumor progression [48]. It is possible that there is a higher expression of pro-apoptotic proteins in low-grade gliomas to synergize with the pro-apoptotic effects of NIX. Compared to the pro-apoptotic role of NIX, NIX-mediated autophagy seems to have a more defined role in glioblastoma. NIX-mediated autophagy in glioblastoma induces cell death via atypical apoptotic pathways. Compared to protective autophagy, over-activated mitophagy leads to autophagy-dependent cell death (ADCD) through multiple pathways. ADCD is a form of regulated cell death characterized by excessive or dysregulated autophagy, leading to the degradation of essential cellular components and ultimately resulting in cell death [49]. This provides a new therapeutic idea for glioblastoma, which are insensitive to apoptosis by classical pathways. AT-101 induces autophagy-dependent cell death and concurrently upregulates the mitophagy receptors BNIP3 and BNIP3L to cause early mitochondrial dysfunction, inducing ADCD development [30,50] (Table 1). In glioblastoma cells, Pitavastatin or Deapioplatycodin D triggered incomplete mitophagy via BNIP3L/NIX. Concurrently, these agents inhibited proliferation by disrupting autophagosome-lysosome fusion, blocking late autophagic flux, and exacerbating mitochondrial damage and accumulation. These findings underscore the potential of exploiting NIX-mediated mitophagy for GBM treatment [51,52] (Table 1). Furthermore, NIX-mediated autophagy is involved in GBM invasive migration, cellular oxidative stress, and tumor stemness maintenance. Because the most aggressive phenotype is one of the hallmarks of glioblastoma. On the one hand, autophagy interacts with the tumor microenvironment to alter the cytoskeleton and reduce cell adhesion, promoting tumor invasion; on the other hand, mitophagy, through alterations in mitochondrial dynamics, provides an energetic basis for tumor migration. SFN-Cys treatment reduced α-tubulin levels, subsequently downregulating NIX interaction with LC3. This impairment of mitochondrial autophagosome formation and selective mitophagy ultimately suppresses GBM cell migration and invasion [53]. In addition, some hypoxic regions formed by the rapid proliferation of highly malignant glioblastomas induce hypoxia-related gene expression, which is one of the reasons for the high expression of NIX. NIX-dependent mitophagy induced by hypoxia and oxidative stress is mediated through the NRF2 transactivation pathway. Ablation of NIX attenuated glioblastoma cell survival by impairing mitochondrial ROS scavenging, disrupting cancer stem cell (CSC) maintenance, and dampening hypoxic HIF/mTOR/RHEB signaling. Importantly, BNIP3 acts as a negative regulator of RHEB expression under both normoxia and hypoxia [48]. This contrasts with the prevailing view that NIX acts synergistically with BNIP3 [15,54]. Other experiments have also demonstrated that NIX and BNIP3 have different effects on glioblastoma under specific conditions [50,55].

**Figure 5:**
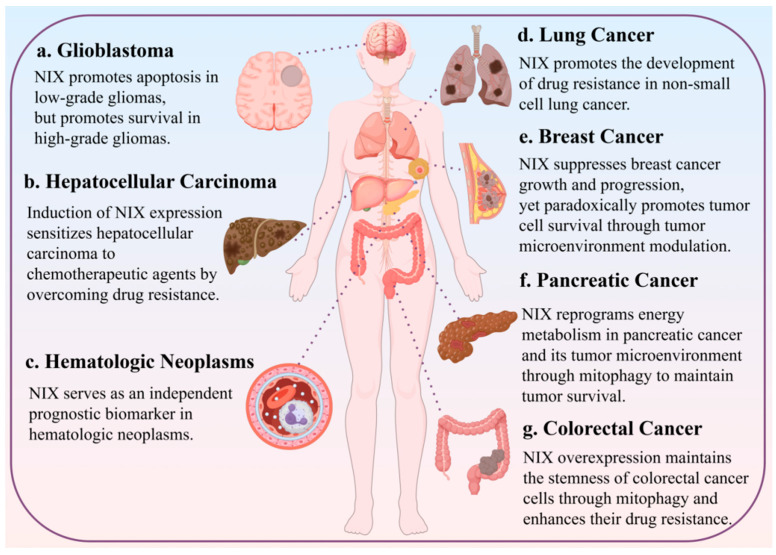
The role of NIX/BNIP3L in tumors.

In summary, the role of NIX in glioblastoma is grade-dependent and exhibits functional duality, transitioning from a tumor-suppressive to a tumor-promoting function with increasing glioma grade. This shift is associated with the progressive loss of apoptotic proteins and the activation of the NF-κB pathway [48]. In glioblastoma, NIX counteracts oxidative stress and maintains tumor stemness through autophagy. Consequently, blocking NIX-mediated protective autophagy represents a potential therapeutic strategy for high-grade gliomas (Figure 5).

### 6.2. Lung Cancer

Lung cancer, a malignant neoplasm originating from the bronchial mucosa, glands, and alveolar epithelium, represents the leading cause of global and Chinese cancer-related mortality. Non-small cell lung cancer (NSCLC) constitutes the predominant histological subtype, accounting for approximately 85% of all pulmonary malignancies [56,57]. NIX-mediated mitophagy promotes drug resistance in NSCLC. Cisplatin, a first-line chemotherapeutic agent for NSCLC, faces significant clinical challenges due to acquired resistance. Research reveals that lung adenocarcinoma upregulates NIX expression through RORA transcription factor binding to the NIX promoter, enhancing autophagic flux and thereby establishing chemoresistance to cisplatin [58]. Meriem Hasmim et al. demonstrated that NANOG, a homeodomain transcription factor critical for maintaining embryonic stem cell pluripotency and promoting tumor progression/immune evasion, induces autophagy during hypoxic stress through direct binding to the BNIP3L promoter. This mechanism facilitates autophagy activation in hypoxic tumor cells and enhances their resistance to cytotoxic T lymphocyte (CTL)-mediated killing [59]. Notably, α-tubulin knockdown decreased NIX and BNIP3 expression while increasing LC3-II/I conversion. Importantly, SFN diminishes both the interaction and colocalization of α-tubulin with NIX. Notably, in NSCLC, NIX-mediated mitophagy can exert therapeutic effects. AM-101, a synthetic benzodiazepine-type GABA(A) receptor activator, enhances mitochondrial clearance by facilitating GABARAP-NIX complex formation and promoting NIX homodimerization, thereby triggering lethal autophagy with anti-tumor activity [60] (Table 1). Furthermore, SFN attenuated the NIX-LC3 II/I interaction and reversed CCCP-induced fatty acid accumulation. These findings suggest that SFN promotes apoptosis through suppression of microtubule-dependent mitophagy [61] (Table 1). On the other hand, inhibition of autophagy promotes gemcitabine-induced NIX-mediated apoptosis [62] (Table 1). NIX cooperates with apoptotic molecules such as activated Caspase 3, Bax, and BNIP3 to jointly drive the apoptotic process. Experiments by Zhou Q et al. demonstrated that the expression of NIX is negatively regulated by ROR1 (Receptor tyrosine kinase-like orphan receptor 1). It participates in autophagy activation via the Akt/GSK-3α/β/mTOR signaling pathway and acts in coordination with cell cycle and apoptosis pathways to jointly regulate the malignant biological behavior of lung adenocarcinoma cells. The upregulation of NIX is one of the key mechanisms by which the malignant phenotype of lung cancer cells is suppressed following ROR1 silencing [43].

Furthermore, the latest research demonstrates that NIX can function through a non-classical nuclear regulatory mechanism in lung adenocarcinoma cells. Under hypoxic conditions, HIF1A, acting as a transcription factor in tumor cells, directly binds to the NIX promoter and upregulates its expression. Subsequently, NIX translocates from the cytoplasm to the nucleus, where it forms a complex with the transcription factor YIN-YANG-1 (YY1) and enhances YY1 binding to the Plasminogen Activator, Urokinase (PLAU) promoter, thereby promoting PLAU transcription and secretion. The secreted PLAU binds to (Plasminogen Activator, Urokinase Receptor) PLAUR on the surface of cancer-associated fibroblasts (CAFs), activating the Hippo-YAP signaling pathway, which drives CAF activation, collagen synthesis, and deposition, and ultimately shapes an immunosuppressive tumor microenvironment (characterized by impaired CD8^+^ T-cell infiltration). This cascade leads to immunotherapy resistance and poor prognosis in lung adenocarcinoma (LUAD) patients. Upamostat can block this pathway, remodel the tumor microenvironment, and enhance the efficacy of PD-1 inhibitors. Importantly, NIX can directly enter the nucleus, breaking the traditional view of its role solely as a mitochondrial membrane protein and establishing it as a critical mediator of hypoxia-induced immunotherapy resistance in lung adenocarcinoma. This highlights the potential value of NIX as a therapeutic target to overcome immunotherapy resistance in LUAD [63].

In summary, NIX enhances tumor resistance to chemotherapeutic agents in non-small cell lung cancer by directly regulating mitophagy and participating in the tumor microenvironment. Furthermore, under hypoxic conditions, NIX can translocate to the nucleus and activate the Hippo–YAP signaling pathway, thereby fostering an immunosuppressive tumor microenvironment, which is detrimental to cancer treatment and prognosis (Figure 5).

### 6.3. Hepatocellular Carcinoma

Liver cancer, a malignant tumor originating in the liver, ranks among the top three causes of cancer-related mortality in 46 countries/regions and is listed among the top five causes in 90 countries/regions worldwide. In China, it is the fifth most commonly diagnosed malignancy and the second leading cause of cancer-related death. Hepatocellular carcinoma (HCC), the most prevalent form of liver cancer, accounts for over 85% of liver cancer incidence. Acquired drug resistance represents a hallmark of HCC disease progression during therapy [56]. The liver’s absence of pain receptors means advanced-stage liver cancer often becomes symptomatic only through compression of surrounding tissues, making late-stage intervention a persistent clinical challenge requiring novel therapeutic strategies. Currently, the primary clinical approaches for treating HCC involve two conventional modalities: surgical intervention and chemotherapy. Although chemotherapy can provide temporary symptomatic relief, the majority of patients experience disease relapse shortly after treatment cessation. It has unsatisfactory outcomes [56]. Zhou S et al. demonstrated that human menstrual blood-derived stem cells (MenSCs) overcome sorafenib resistance in HCC by inducing NIX expression through promoter demethylation. This epigenetic reprogramming shifts protective autophagy toward lethal autophagy, thereby restoring therapeutic efficacy against advanced HCC [64] (Table 1). Similarly, Calvisi DF et al. found that NIX and BNIP3 promoter methylation was associated with poor prognosis in HCC [65]. Furthermore, platycodin D2 (PD2) induces NIX-mediated mitophagy in HCC cells, activating the p21/cyclin A2 pathway to promote cellular senescence. This senescence exerts dual anti-tumor effects: as a typically irreversible process, it directly suppresses HCC progression. Concurrently, senescent cells develop a pro-inflammatory secretory phenotype that actively stimulates immune responses and recruits immune cells, ultimately improving therapeutic resistance in liver cancer [66] (Table 1). As established, oxidative phosphorylation (OXPHOS) generates ATP with significantly higher efficiency than glycolysis. Consequently, even moderate impairment of mitochondrial respiration necessitates a substantial upregulation of glycolytic flux to maintain cellular energy homeostasis [67].

NIX-mediated mitophagy additionally reprograms energy metabolism in HCC cells, shifting cellular energy production from OXPHOS toward anaerobic glycolysis. This metabolic adaptation maintains cancer stemness properties, which may potentially compromise the therapeutic efficacy of treatments targeting HBx (the multifunctional protein encoded by the hepatitis B virus) in HBV-associated HCC [68]. All of the above studies have elucidated that NIX-dependent mitophagy has a positive effect on ameliorating drug resistance in HCC. Furthermore, BNIP3L, which plays an important role in the autophagic and apoptotic cell death, is the target molecule in HCC treatment. Multiple studies demonstrate that apoptin treatment elevates ROS levels in liver cancer cells, triggering mitochondrial depolarization and subsequent induction of intrinsic apoptosis and NIX-mediated mitophagy [59,60] (Table 1). Intriguingly, although Li et al. confirmed that reactive oxygen species (ROS) serve as the critical nexus connecting autophagy and apoptosis in HCC cells, NIX-mediated mitophagy paradoxically suppresses apoptotic activation in this context [69]. Elevated ROS levels activate the NIX-mediated apoptotic pathway by disrupting ΔΨm, triggering Cytc release and subsequent apoptosis. Conversely, ROS accumulation simultaneously induces mitophagy, which reduces ROS generation through mitochondrial clearance, thereby suppressing apoptotic initiation [70]. Precisely modulating the equilibrium between NIX-mediated cytoprotective autophagy and pro-apoptotic signaling represents a pivotal therapeutic strategy for HCC (Figure 4).

In summary, demethylation of the NIX promoter may contribute to the treatment of drug-resistant hepatocellular carcinoma. However, NIX-mediated mitophagy promotes the progression of chemoresistance by clearing ROS and facilitating metabolic reprogramming for energy production. Therefore, beyond depending on the molecular subtype of hepatocellular carcinoma, whether NIX ultimately promotes apoptosis is also determined by the extent of the mitophagy it initiates (Figure 5).

### 6.4. Breast Cancer

NIX, as a mitochondrial autophagy-selective receptor, targeting NIX therapy inhibits breast cancer cell growth, metastasis, and invasion. In female nude mice bearing orthotopic triple-negative breast cancer (TNBC) xenografts, decreased expression of autophagy-related proteins, including NIX, was observed, and high-intensity interval training (HIIT) restored NIX-mediated autophagic flux and suppressed tumor growth through AMPK/ULK1-dependent mechanisms [71]. Huang L et al. demonstrated that triptolide-induced autophagy inhibits proliferation, metastasis, and invasion of human breast cancer cells through ATG5-dependent mitophagic flux enhancement and FAK/Src signaling disruption [72] (Table 1). In HER2-overexpressing breast cancer cells of MDA-MB-453, the acid-ground nano-realgar processed product (NRPP) activates the p53/BNIP3/NIX mitophagy axis, suppressing tumor growth both in vitro and in vivo through dual-phase mitochondrial clearance [73] (Table 1). In contrast, NIX exerts a protective role through autophagy in hormone receptor-positive breast cancer. Experiments by Mauro-Lizcano M et al. demonstrated that NIX-mediated mitophagy enhances mitochondrial activity in hormone receptor-positive (Luminal A) breast cancer, thereby providing tumor cells with increased ATP, sustaining tumor stemness, and promoting proliferation and migration [74]. This distinct effect may be attributed to the fact that in this breast cancer subtype, NIX-mediated mitophagy does not lead to a reduction in mitochondrial mass, which differs from its role in HER2-positive and triple-negative breast cancers. Similarly, experiments by Lyons A et al. showed that in hormone receptor-positive breast cancer, Insulin-like Growth Factor 1 (IGF-1) induces BNIP3/NIX-dependent mitophagy, which helps sustain cancer cell viability and contributes to chemoresistance [75]. These findings illustrate that NIX-mediated autophagy plays fundamentally divergent roles across different molecular subtypes of breast cancer, closely tied to whether the process balances or disrupts mitochondrial mass and activity.

NIX-mediated apoptosis and mitophagy interact to influence breast cancer therapy. NIX, an apoptotic protein, would have lower expression in breast cancer tissues compared to healthy tissues [76]. Recent studies demonstrate that a recombinant fragment of human κ-Casein (RL2) induces mitophagy, resulting in significant ATP depletion. The cytotoxic mechanism of RL2 was characterized by three hallmark molecular events: (1) suppression of adenosine triphosphate (ATP) generation; (2) accumulation of LC3B-II; (3) upregulation of mitophagy receptors BNIP3 and NIX. Additionally, the activity of phosphatase and PINK1 was also observed. In TNBC, RL2-induced NIX-mediated mitophagy inhibits tumor necrosis factor-related apoptosis-inducing ligand (TRAIL)-induced apoptosis in the short term, but prolonged co-stimulation of RL2 increased cellular sensitivity to TRAIL-induced cell death, which is in part related to ATP depletion by mitochondrial autophagy [77] (Table 1). Likewise, in TNBC, RL2 has been demonstrated to induce mitophagy, including NIX-mediated pathways, and to synergize with doxorubicin (DXR) in promoting apoptosis [78] (Table 1). Zhou S et al. demonstrated that pharmacological inhibition of EGFR or HER2 selectively upregulates BNIP3L, a critical mediator of chemosensitization in breast cancer cells. Notably, their findings revealed an inverse correlation between BNIP3L expression and EGFR levels in clinical breast cancer specimens [79]. Anoikis, as a specialized form of apoptosis triggered by cell detachment from the extracellular matrix or loss of intercellular adhesion, is suppressed during epithelial carcinoma metastasis. Breast cancer cells downregulate KDM3A (a histone demethylase), which inhibits its recruitment of NIX [80], thereby reducing anoikis to facilitate tumor growth [81]. While these studies collectively suggest the indispensable role of NIX in breast cancer chemotherapy, other evidence indicates that in TNBC, NIX may counteract FBP1 (fructose-1,6-bisphosphatase 1)-induced apoptosis by activating Beclin 1-dependent mitophagy to scavenge ROS [82].

In contrast to the Warburg effect observed in most malignancies, human breast tumors frequently exhibit transcriptional upregulation of mitochondrial OXPHOS. Consequently, enhanced OXPHOS activity represents a distinguishing metabolic feature of breast cancer epithelial cells. Scholars propose a novel metabolic paradigm—the Reverse Warburg Effect—to resolve longstanding contradictions in conventional tumor metabolism models. In this framework, oxidative stress induces fibroblast autophagy and mitophagy, driving anaerobic glycolysis through mitochondrial impairment. The resulting glycolytic phenotype promotes L-lactate secretion, which epithelial cancer cells utilize as an energy source, establishing a parasitic metabolic symbiosis between stroma and tumor [83]. Cathepsin B, BNIP3L, and MCT4 are rarely expressed in tumor cells, a phenomenon that may also contribute to the enhanced OXPHOS activity of breast cancer cells. In addition, activation of HIF-1α or NFκB transcription factors induced by hypoxia, oxidative stress, and other adverse factors in the stromal region of breast cancer can easily induce the expression of NIX and autophagy in stromal cells such as CAFs [84]. Furthermore, reduced Caveolin-1 expression and diminished mitochondrial mass promote a shift toward aerobic glycolysis in these cells, providing metabolic precursors to support OXPHOS in breast cancer. These metabolic precursors fuel adjacent OXPHOS-dependent cancer cells and simultaneously confer apoptosis resistance on these cells [85]. Intriguingly, HIF-1α overexpression-induced autophagy unexpectedly suppresses tumor growth in breast cancer cells, whereas this process occurs independently of NIX expression and mitophagy activation [85]. In addition, TNBC cells also provide ITGβ4 protein (Integrin Subunit Beta 4, Integrin Beta 4) to CAFs via exosomes, which induces c-Jun phosphorylation to promote BNIP3L-dependent mitochondrial autophagy and lactic acid production, a metabolic reprogramming detrimental to the prognosis of breast cancer patients [36].

In summary, while NIX is generally lowly expressed in breast cancer cells, the therapeutic implications of its mediated mitophagy are highly dependent on the molecular subtype. In triple-negative and HER2-overexpressing breast cancers, restoring NIX function and inducing mitophagy may cooperate with other apoptotic proteins to enhance chemosensitivity. However, in Luminal A breast cancer, NIX-mediated mitophagy paradoxically protects tumors, likely because it does not lead to a net loss of mitochondrial mass in this context. Furthermore, the distinct expression pattern of NIX—being highly expressed in the tumor microenvironment but low in tumor cells—facilitates a metabolic coupling. Through mitophagy, cancer-associated fibroblasts (CAFs) in the microenvironment undergo aerobic glycolysis, while the cancer cells themselves maintain high oxidative phosphorylation. This “Reverse Warburg Effect,” where glycolytic metabolites from the stroma fuel mitochondrial respiration in cancer cells, supports tumor proliferation and is associated with poorer patient prognosis (Figure 5).

### 6.5. Pancreatic Cancer

Pancreatic cancer is a malignant tumor originating from the ductal epithelium and acinar cells of the pancreas. Pancreatic ductal adenocarcinoma (PDAC) is the most prevalent subtype, accounting for over 90% of cases [86]. PDAC is characterized by features such as hypovascularity, nutrient deprivation, high malignancy, rapid proliferation, difficulty in early diagnosis, and a dismal prognosis, earning it the designation as one of the most lethal malignancies. Globally and in China, pancreatic cancer ranks as the sixth leading cause of cancer-related mortality [87]. The study by Humpton TJ et al. proposed that Kirsten rat sarcoma viral oncogene homolog (KRAS) stimulates NIX expression via activation of the MAPK pathway through its downstream effector mitogen-activated protein kinase (MEK). Furthermore, NIX expression was found to be positively correlated with the malignancy of pancreatic cancer [88]. Compared to normal tissue, NIX expression shows progressive upregulation in pancreatic cancer. Genetic ablation of NIX significantly attenuates PDAC progression and extends survival in KPC mouse models. This tumor-suppressive effect correlates with mitochondrial functional restoration, elevated NADPH demand, and impaired proliferative capacity under glucose deprivation [88]. Lidamycin (LDM), an enediyne antibiotic derived from Streptomyces metabolites, exhibits potent anti-tumor activity. In pancreatic cancer cells, LDM upregulates mitofusin-2 (Mfn2) expression, subsequently inhibiting cellular migration and invasion. Importantly, Mfn2 knockdown attenuates LDM’s suppressive effects on tumor cell motility while concurrently impairing NIX-mediated mitophagy [89] (Table 1). In cancer cells, elevated NIX expression reduces functional mitochondria via mitophagy, thereby decreasing oxygen consumption. Concurrently, this metabolic shift suppresses aerobic oxidation and redirects flux predominantly through the non-oxidative phase of the pentose phosphate pathway (PPP). This reprogramming maintains the NADPH/NADP^+^ ratio [87], ultimately stabilizing intracellular redox homeostasis. Otherwise, NIX acts as an apoptotic protein and also activates the apoptotic pathway in pancreatic cancer cells. Octyl gallate (OG) induces HIF-1α accumulation, thereby promoting NIX expression. This enhanced NIX level strengthens its interaction with anti-apoptotic proteins Bcl-2 and Bcl-XL, triggering downstream oligomerization of BAK and BAX. Consequently, this oligomerization alters mitochondrial membrane permeability (MMP), leading to the release of Cytc and other pro-apoptotic factors, ultimately initiating the apoptotic cascade [90] (Table 1), which further highlights the critical importance of the NIX-mediated apoptotic pathway for pancreatic cancer treatment (Table 1).

In summary, NIX expression progressively increases with higher tumor grade in pancreatic cancer, which is associated with the activation of the MAPK signaling pathway. Overexpressed NIX reprograms the energy metabolism of cancer cells through mitophagy, maintaining the NADPH/NADP^+^ ratio via the pentose phosphate pathway. Within the tumor microenvironment, NIX also promotes tumor migration by facilitating NADPH accumulation through the activation of NADK2 (Figure 5).

### 6.6. Colorectal Cancer

Colorectal cancer (CRC) is a malignant tumor originating from the mucosal epithelium of the colon and rectum, with adenocarcinoma being the predominant pathological type, accounting for approximately 90–95% of all cases. CRC ranks as the third most commonly diagnosed malignancy globally [57]. In China, it has become the second most common cancer after lung cancer, exhibiting a persistently rising age-standardized incidence rate and a trend toward affecting younger populations [56]. Furthermore, CRC shows geographical disparities characterized by a higher incidence in high-income regions and a faster-growing mortality rate in low-income areas. These variations may be closely associated with genetic predisposition, lifestyle factors, and intestinal diseases.

The expression of NIX is significantly higher in CRC tissues compared to normal colorectal mucosa. Studies indicate that high NIX expression is significantly correlated with poor differentiation in CRC. Furthermore, CRC patients with high NIX expression exhibit reduced overall survival, suggesting NIX as a potential biomarker for poor prognosis [91]. Under hypoxic conditions, HIF-1α induces NIX expression. NIX-mediated mitophagy helps maintain cancer stem cells (CSCs) and enhances chemoresistance. Specifically, hypoxia-induced NIX overexpression enhances autophagy, subsequently activating the PRKC-EZR signaling axis, which ultimately sustains CSC populations and their self-renewal capacity [92]. Experiments by Yan C et al. also demonstrated that NIX is specifically overexpressed in colorectal CSCs. The mitophagy mediated by NIX protects CSCs from chemotherapy-induced damage by doxorubicin (DXR). Knockdown of NIX specifically blocks mitophagy in CSCs, reverses their resistance to DXR, and thereby increases chemotherapy sensitivity in CRC [93]. 5-Fluorouracil (5-FU) is a cornerstone chemotherapeutic agent for CRC. Research has found that combining 5-FU with chloroquine significantly downregulates NIX expression. This combination not only inhibits the malignant phenotype of colon cancer cells but also enhances their immunogenicity, promoting anti-tumor immune responses, ultimately contributing to improved therapeutic outcomes [94]. Additionally, in CRC cells, overexpression of GPR176, a member of the G protein-coupled receptor superfamily, can phosphorylate NIX via the GPR176/GNAS/cAMP/PKA axis, leading to a loss of its mitophagic function [95].

In CRC, NIX cooperates with p53 to promote apoptosis. In a subset of CRC cases with wild-type p53, NIX, along with genes such as DR5, Fas, and Bax, mediates the apoptotic response following DNA damage induced by chemotherapeutic agents like 5-FU and ADR [96]. The experiment by Wilfinger N et al. further demonstrated that NIX is a core molecule in the response of p53 wild-type CRC cells to KP46 (tris(8-quinolinolato)gallium(III), a gallium-based anticancer drug) [97]. KP46-induced intracellular iron and heme deficiency leads to the nuclear accumulation of p53, which transcriptionally upregulates NIX. Subsequently, p53 and NIX cooperatively regulate mitochondrial permeability transition (MPT), ultimately inducing cancer cell death.

In summary, under hypoxic conditions, NIX is induced by HIF-1α. It mediates mitophagy to clear ROS, thereby maintaining the self-renewal of CSCs and enhancing chemoresistance. However, in CRC with functional p53, NIX can cooperate with other pro-apoptotic proteins and modulate the mitochondrial permeability transition pore to promote apoptosis (Figure 5).

### 6.7. Hematologic Neoplasms

Unlike solid tumors originating from discrete organs or tissues, hematological malignancies arise from hematopoietic or lymphoid systems, exhibiting diffuse infiltration and poor detectability by conventional imaging. The absence of circumscribed masses precludes surgical resection, making systemic clearance of malignant cells—via pharmacological or immunotherapeutic interventions—and restoration of normal hematopoiesis the central therapeutic objectives. Leukemia ranks as the 10th leading fatal cancer globally [57], predominantly affecting the elderly while also representing the most prevalent childhood malignancy worldwide and the highest-incidence cancer among Chinese children aged 0–14 years [98]. NIX serves as an independent prognostic indicator in myelodysplastic syndromes (MDS), which is a heterogeneous group of clonal hematopoietic stem cell disorders with high risk of progression, now classified as a preleukemic condition. Multiple studies demonstrate suppressed NIX expression in MDS bone marrow samples, attributable to aberrant NIX methylation. As disease advances, progressive NIX downregulation correlates with aggravated symptoms and poor prognosis, establishing its independent prognostic value [99,100,101]. Notably, MDS patients with high HIF-1α exhibit enhanced sensitivity to standard therapy 5-azacitidine (5-AZA) and prolonged survival, concomitant with elevated NIX expression—further validating NIX’s protective role [74].

Analogous to MDS, acute myeloid leukemia (AML) patients show marked NIX reduction that intensifies with disease progression [75,101]. Like most malignancies, AML exhibits an imbalance in the BCL-2 family: pro-apoptotic members (including NIX) are suppressed. Pharmacologically restoring pro-apoptotic proteins (e.g., NIX) while inhibiting anti-apoptotic counterparts (Bcl-2, BCL-XL) represents a viable therapeutic strategy [102,103]. Intriguingly, the hypomethylating agent decitabine (DAC) differentially regulates NIX and BNIP3. Both are epigenetically silenced in AML but rescued by DAC. Unlike BNIP3—a direct DAC target [104], NIX silencing paradoxically enhances DAC-induced apoptosis without modulating autophagy or Bcl-2 expression [75,101]. Functioning as an autophagy effector, NIX contributes to AML pathophysiology. Unlike normal cells, AMLs exhibit features such as deletions in autophagy gene loci and reduced autophagy gene expression [105], increased mitochondrial mass [106], enhanced OXPHOS capacity [107], and diminished spare respiratory capacity (SRC) [108]. Patients with high mitophagy and low glycolysis have a good prognosis [109], reinforcing NIX’s prognostic utility. Mitochondrial-targeted agents preferentially induce AML cytotoxicity [80,110], and NIX deficiency heightens AML cell sensitivity via disrupted mitochondrial membrane potential, ROS accumulation, and dysregulation of oxidative stress [9,111]. NIX knockdown in AML cells further impairs mitophagy, reducing SRC and antioxidant defense. This mitophagy defect synergizes with fatty acid oxidation (FAO) inhibitors to disrupt mitochondrial respiration, culminating in ATP depletion and cell death [112].

In erythroleukemia, heme (a physiological erythroid maturation stimulus) activates NIX-mediated mitophagy to promote red blood cell maturation—a mechanism that can be exploited for chronic myeloid leukemia therapy [113]. In multiple myeloma, NIX expression is significantly lower in patient marrow than in healthy donor marrow, showing an inverse correlation with risk factors (β2-microglobulin, creatinine) and a positive correlation with protective factors (albumin, hemoglobin) [114,115,116], highlighting NIX’s therapeutic potential across hematologic malignancies (Figure 4).

In summary, NIX can serve as a prognostic indicator for various hematological malignancies. Its expression is often suppressed during tumor progression, which typically reflects defects in apoptotic proteins, alterations in cellular autophagy, and is accompanied by reduced mitophagy alongside enhanced oxidative phosphorylation. These factors collectively contribute to poor prognosis in AML (Figure 5).

**Table 1:** NIX in Tumors and Its Role in Anti-tumor Pharmaceuticals.

Tumors	Pharmaceutical	Physiological Functions and Disease Links	References
Glioblastoma	Sulforaphane	SFN induced apoptosis via NIX-mediated mitophagy.	[61]
	Sulforaphane-cysteine	SFN-Cys lowered the expression of NIX.	[53]
	Deapioplatycodin D	BNIP3L-mediated mitophagy was induced by Deapioplatycodin D to inhibit Glioblastoma cell growth.	[52]
	Pitavastatin	Pitavastatin induces NIX-mediated mitochondrial autophagy and promotes cell death when combined with temozolomide.	[51]
	AT 101	AT 101 stimulates the induction of BNIP3 and BNIP3L, as well as HMOX1, which further amplify mitophagy and partially contribute to AT 101’s toxicity in glioma cells.	[50]
Lung cancer	Cynomorium coccineum	In lung cancer cells, AM-101 modulates the GABA(A) receptor, leading to selective autophagy through GABARAP oligomerization and stabilization of the mitochondrial receptor NIX.	[117]
	AM101	In lung cancer cells, AM-101 modulates the GABA(A) receptor, leading to selective autophagy through GABARAP oligomerization and stabilization of the mitochondrial receptor NIX.	[60]
	Gemcitabine	Gemcitabine treatment significantly increased BNIP3L expression in Human Lung Cancer Cells.	[62]
Hepatocellular carcinoma	Cynomorium coccineum	Cynomorium coccineum can activate and enhance the expression of the mitochondria-associated cell death proteins BNIP3 and BNIP3L, thereby triggering apoptosis.	[117]
	Platycodin D2	PD2 induced mitophagy via NIX, leading to cell senescence through the P21/Cyclin A2 signaling pathway.	[66]
	Sorafenib	Sorafenib resistance in human menstrual blood-derived stem cells is suppressed through NIX-dependent mitophagy activation.	[64]
	Polygalacin D	Polygalacin D inhibits the proliferation of hepatocellular carcinoma cells via pathways involving BNIP3L-dependent mitophagy and intrinsic apoptosis.	[118]
Breast cancer	Triptolide	Triptolide suppresses the migration and invasiveness of MDA-MB-231 cells by promoting NIX expression.	[72]
	Acid ground nano-realgar processed product, NRPP	NRPP inhibited breast cancer cells’ growth by inducing mitophagy via the p53/BNIP3/NIX pathway.	[73]
	Recombinant lactaptin 2 combined with doxorubicin	Recombinant lactaptin 2 caused an upregulation of BNIP3L/NIX in breast cancer cells.	[78]
	Recombinant lactaptin 2 combined with TRAIL	Recombinant lactaptin 2 acts via the upregulation of NIX, leading to the loss of adenosine triphosphate production.	[77]
Pancreatic ductal adenocarcinoma	Lidamycin	Lidamycin activates EMT by inducing the BNIP3L-mediated mitophagy in pancreatic cancer cells.	[89]
	Octyl gallate	Octyl gallate elevates BNIP3L levels.	[90]
Colorectal cancer	5-FU combined with CQ	Combined treatment of colon cancer cells with 5-fluorouracil (5-FU) and the autophagy inhibitor chloroquine (CQ) downregulates NIX mRNA by blocking the autophagy pathway.	[94]
	KP46	KP46 activates p53 via iron depletion, which, in turn, transcriptionally upregulates NIX. As a key downstream effector, NIX subsequently mediates the cytotoxic effects of KP46 in p53-wild-type colorectal cancer cells by promoting PARKIN-dependent mitophagy and sensitizing the mitochondrial permeability transition (MPT).	[97]
Hematologic neoplasms	Cereblon modulator CC-885	CC-885 regulates BNIP3L, which depends on mitophagy for E3 ligase-dependent ubiquitination and degradation.	[9]

## 7. Conclusions 

In tumor cells, NIX/BNIP3L-mediated mitophagy constitutes a core regulatory network that intricately interacts with apoptosis and oxidative stress. Under stress conditions such as hypoxia, the accumulation of reactive oxygen species (ROS) generated by damaged mitochondria, coupled with the upregulation of NIX expression, cooperatively initiates mitophagy. Moderate levels of mitophagy, by promptly clearing dysfunctional mitochondria and the associated ROS, effectively inhibit apoptosis and confer a survival advantage to tumor cells. In contrast, excessive mitophagy or autophagic cell death may exacerbate ROS production through mechanisms such as cytochrome c release, thereby promoting apoptosis. Furthermore, NIX-mediated mitophagy helps tumor cells maintain redox homeostasis by modulating mitochondrial oxidative phosphorylation, activating antioxidant systems, and inducing metabolic reprogramming, while simultaneously counteracting apoptosis triggered by endoplasmic reticulum stress induced by excessive ROS. Notably, ROS released during the apoptotic process can further activate autophagy and oxidative stress, forming a dynamic and complex feedback network of crosstalk. In summary, the NIX-mitophagy-ROS signaling axis represents a crucial mechanism through which cells monitor and repair mitochondrial quality to balance survival and death. Within the tumor context, this axis exhibits a significant, context-dependent duality: it can serve as a protective mechanism, enabling tumor cells to adapt to the microenvironment and resist apoptosis; yet, under specific conditions, it can also transform into a pro-death pathway. Therefore, a deeper dissection of the dynamic functional patterns of this pathway across different tumor types and stages of progression holds substantial theoretical value and clinical promise for developing novel anti-tumor strategies targeting mitophagy and oxidative balance.

## Data Availability

Data sharing is not applicable. No new data were created or analyzed in this study.

## Abbreviations

The following abbreviations are used in this manuscript:

BAK	BCL2-antagonist/killer 1
BAX	BCL2-associated X protein
Bcl-2	B-cell lymphoma/leukemia-2
Bcl-XL	B-cell lymphoma-extra large
PRKN	E3 ubiquitin-protein ligase parkin
FUNDC1	FUN14 domain-containing protein 1
